# The effect of push frequency and stroke time on wheelchair maneuverability among wheelchair tennis athletes on hard tennis court

**DOI:** 10.1038/s41598-024-83127-7

**Published:** 2024-12-28

**Authors:** Ajitkumar Esak, Nur Azah Hamzaid, Edelene See, Selina Khoo

**Affiliations:** 1 Department of Biomedical Engineering, Faculty of Engineering, Universiti Malaya, 50603 Kuala Lumpur, Malaysia; 2 Faculty of Sports and Exercise Science, Universiti Malaya, 50603 Kuala Lumpur, Malaysia

**Keywords:** Wheelchair athletes, Wheelchair tennis, Push frequency, Stroke time, Propulsion, Biological physics, Biomedical engineering

## Abstract

Wheelchair propulsion is a fundamental skill in wheelchair sports, particularly in wheelchair tennis. To achieve optimal mobility during wheelchair athletic performance, it is essential to consider propulsion techniques. This study examines the effect of push frequency and stroke duration on wheelchair maneuverability, measured by velocity during propulsion, among wheelchair tennis athletes. The athletes (*N* = 9; 5 elite and 4 novice) performed three tests; namely the comfortable speed propulsion test, sprint test and round-trip test; with and without holding their racquet, over their hard court. Results revealed that push frequency had positive correlation with propulsion velocity (*r* = 0.840, *p* < 0.001) while stroke time was negatively correlated with velocity (*r* = -0.859, *p* < 0.001). Propulsion performance between elite and novice wheelchair athletes, and between propelling with and without racquet were also reflected through these parameters. The rate of perceived exertion (RPE) had significantly positive but low correlation with velocity and push frequency, and significant negative correlation with stroke time. This study could serve as a recommendation for wheelchair tennis athletes and coaches in planning their training protocols and techniques.

## Introduction

Wheelchair maneuverability in wheelchair tennis contributes to quick movements and sprints across the court, thus become an important advantage for the athletes^1^. While the wheelchair design, dimension and proportions itself contributes to better ability of the athletes to move around the court, in particular the height of backrest in the wheelchair^2^, the wheel configuration^3^, its rolling resistance^4,5^, and wheelchair camber angles^6^ ; the biomechanical behavior and techniques of the athletes play a significant role in gaining their competitive edge.

Various contributors towards superior maneuverability of wheelchair include the push frequency, which refers to the number of pushes per time performed by the wheelchair athletes. Increasing push strength will increase the acceleration per push^1^, and consequently propulsion speed increases with the oxygen uptake, i.e. VO_2,_ based on a long-term training study among international female wheelchair tennis players^7^.

The push frequency is often considered to be among the most dominant contributors towards the velocity of the propulsion. Mason et al. found that push frequency and propulsion speed were positively correlated, however, at higher reverse speeds the physiological propulsion demand increases^8^. Goosey-Tolfrey and Kirk (2003) reported that the push frequency correlates positively with the blood lactate concentration^9^. The level of blood lactate concentration tends to increase with the intensity level of the exercise. The push frequency notably influences the work per push in the opposite direction where decreases in push frequency cause a higher amount of work per push required to propel. In terms of stroke length or stroke time, also referred to as push time, Van Drongelen et al. (2013) concluded that the push time has a negative correlation with propulsion speed when propulsion power is imposed, with a further increase in the push angle over which positive torque was applied^10^.

In terms of effort, the Rate of Perceived Exertion (RPE) was investigated to represent the physiological intensity. Researchers found strong correlation between the measurable cardiorespiratory responses such as heart rate and blood lactate concentration with the participants’ RPE rating^11–13^ but with limited statistical significance when tested upon trained wheelchair sportspersons^14^.

This study aims to investigate the wheelchair maneuverability among wheelchair tennis athletes in ground hardcourt setting, measured through the athletes’ propulsion velocity. The propulsion techniques that were examined are the varying push frequency and stroke time when completing multiple propulsion tests. In particular, this study aims to examine the correlation between propulsion velocity with push frequency and stroke time among wheelchair tennis athletes in their actual training and competition environment. Additionally, this study describes the RPE with respect to the athletes’ different propulsion techniques and conditions.

## Method

### Pilot study participants

To determine the prominent wheelchair propulsion parameters, the study was piloted with 12 non-disabled participants (6 males and 6 females; age 19.9 ± 1.44 years old, BMI = 22.27 ± 4.44). Participation was voluntary and participants provided written informed consent prior to joining the study. The study protocol was conducted in accordance with the ethical standards in the 1964 Declaration of Helsinki, and the experimental protocol were endorsed by the Final Year Project Committee, Department of Biomedical Engineering, Faculty of Engineering Universiti Malaya.

**Fig. 1:**
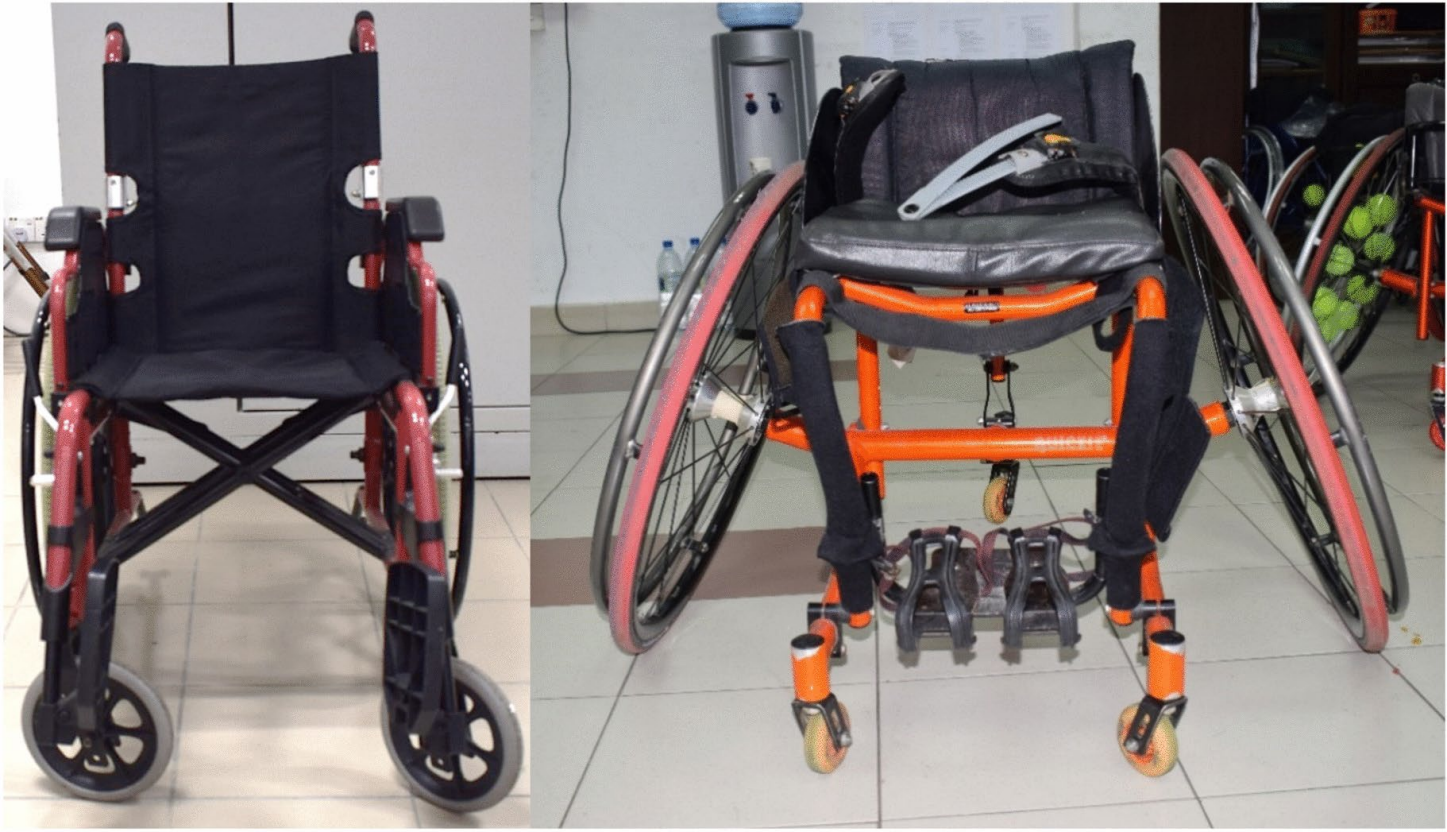
(**a**) Standard manual wheelchair used by non-disabled participants, and (**b**) tennis wheelchair used by wheelchair tennis athletes.

A 22-inch standard manual wheelchair (Fig. 1a: ANI lightweight DAF Quick Release (QR) wheelchair (14kg), 22-inch (14 × 17 inch) seat size, with aluminum frame, flip-back footrest, detachable footrest, 24″ PU MAG rear wheels and 8″ solid castor wheels) was used in this study for the non-disabled participants. All participants were first asked to familiarize themselves with the wheelchair by propelling it for about 10 to 20 min. The participants were taught basic propulsion methods as all of them had little to no experience in using a wheelchair. They were asked to perform manual wheelchair propulsion at their comfortable speed and then sprint tests were conducted on rough, smooth, and ramp surfaces. The non-disabled participants were then allowed to propel using self-selected propulsion technique^15^. The performance of the participants was recorded using a 16- megapixel camera of an Android smartphone. The actual distance of the three types of surfaces were measured using measuring tape for calibration measure of the travelled distance using Kinovea.

### Pilot study protocol and parameters of interest

The non-disabled participants performed normal wheelchair push for the 10s period at their comfortable propulsion speed, on a rough surface, a smooth surface and a ramp. One lap of multiple propulsions was performed on each type of surface. The participants were allowed a minimum of one minute rest between laps.

Then, each participant performed comfortable speed and sprints test for 5s on the rough surface and smooth surface. Two laps of multiple propulsions were performed by the participants on each surface and the average value of all individual propulsion cycle was taken. Again, the participants were given one minute rest period between trials.

The recorded videos of all trials were analyzed through Kinovea software for parameters such as the time and distance. The actual distance of the three tracks was measured using a measuring tape for calibration of the travelled distance using Kinovea. The measured distance was used to calculate the average velocity throughout the propulsion cycle. The average stroke length of each propulsion is calculated as the time between successive push starts. Push frequency refers to the number of pushes performed per second (push/s), while stroke time refers to the time for one complete push (from the push start phase to push end phase) measured in the unit of time (s). Average propulsion velocity is the average distance traveled in unit time (ms^−1^).

The examined parameters of the non-disabled propulsion provided indication about the most relevant parameters for wheelchair maneuverability among the national wheelchair tennis athletes. From our pilot study with the non-disabled group, it was observed that the push frequency and stroke time are user-controllable parameters that have significantly higher impact on wheelchair mobility apart from stroke length. Stroke time was chosen to be examined for the main study instead of the stroke length since the pilot study shows that different propulsion technique or stroke pattern might have a greater influence in variability on the stroke length. Therefore, stroke time was considered to be the better objective measure to represent their propulsion behavior.

### Study protocol with wheelchair tennis athletes

To conduct the main investigation, nine wheelchair tennis athletes (5 elite and 4 novice) from the national wheelchair tennis team volunteered to participate. Their experience in wheelchair tennis sports ranged from 2 months to 6 years (Table 1). The athletes used their own cambered wheel sports wheelchair as illustrated in Fig. 1b. The athletes were also allowed to propel using their self-selected technique^15^ in all trials.

**Table 1:** Description of the wheelchair tennis athletes.

Athlete	Sex	Injury Type	Elite/Novice	Weight (Kg)	Experience in wheelchair tennis
1	Male	SCI	Elite	70	6 years
2	Male	ALD	Elite	58	4 years
3	Male	ALD	Elite	56	3 years 3 months
4	Female	SCI	Elite	54	3 years
5	Male	SCI	Elite	54	2 years
6	Male	SCI	Novice	66	1 year
7	Female	SCI	Novice	60	6 months
8	Male	ALD	Novice	55	6 months
9	Male	SCI	Novice	62	2 months

*SCI Spinal Cord Injury, *ALD* Amputation / Limb difference.

All athletes were asked to perform three types of propulsion activity on standard tennis hardcourt covered with acrylic layer using their tennis wheelchair. A 10m distance of comfortable propulsion speed test, 10m sprint test and 20m round trip sprint test where the athletes sprinted towards the end point of 10m and returned to the initial start point. The athletes were asked to perform the three propulsion trials with and without holding their tennis racquet for three repetitions respectively.

After each trial, the athletes were asked on their rating on rate of perceived exertion (RPE) based on the Borg scale and their response was recorded. The RPE is measured based on the Borg Scale with a range from 0 to 10. The value of 0 indicates that the activity is extremely light and no exertion is felt by the athlete. The maximum value of 10 indicates that the activity is extremely hard and the athletes are exhausted maximally. The published model of Borg RPE scale by Cleveland Clinic was used for explanations to the athletes.

A 3-min interval resting period were given to the athletes in between each trial lap. The tests were conducted before their actual tennis training starts so that any effect on RPE responses due to exertion caused by training activity can be ruled out. The performance of the athletes was recorded using Nikon D5600 DSLR Camera and tripod stand to obtain highly accurate videos with high resolution. Kinovea was used to perform the data extraction and analysis, similar to the non-disabled group. All statistical analysis was performed using the IBM SPSS version 25 software.

## Results

### Pilot trial outcome

From the non-disabled group, push frequency; i.e. the number of pushes per second (push/s), stroke time (s) and average velocity (m/s) were calculated for both comfortable speed propulsion test and sprint test performed in three different types of surfaces (Table 2).

**Table 2:** Push frequency, stroke time and velocity across different surfaces at comfortable and sprint test among non-disabled group.

	Rough	Smooth	Ramp	
Push frequency at comfortable speed propulsion test (push/s)	1.065 ± 0.19	0.985 ± 0.18	0.956 ± 0.12	
Push frequency during sprint test (push/s)	1.437 ± 0.29	1.440 ± 0.28		
Stroke time during comfortable speed test (s)	1.11 ± 0.19	1.20 ± 0.19	1.14 ± 0.18	
Stroke time during sprint test (s)	0.86 ± 0.18	0.83 ± 0.14		
Velocity during comfortable speed propulsion test (m/s)	0.43 ± 0.11	0.47 ± 0.1	0.4 ± 0.12	
Velocity during sprint test (m/s)	0.66 ± 0.14	0.86 ± 0.14		
Pearson’s correlations	Comfortable speed		Sprint	
Push Frequency (push/s) vs Velocity (ms-1)	r = 0.478	(p = 0.003)	r = 0.43	(p = 0.036)
Stroke Length (s) vs Velocity (ms-1)	r = − 0.4	(p = 0.016)	r = − 0.485	(p = 0.016)

It was found that push frequency and stroke time were the two user-input parameters that influenced the wheelchair velocity the most.

### Effect of push frequency and stroke time on wheelchair maneuverability and exertion level measurement among wheelchair tennis athletes

In the wheelchair tennis athletes’ group, the push frequency was also found to have positive correlation with propulsion velocity for both comfortable speed test (Table 3) and sprint test, both one-way (Table 4) and round trip (Table 5). Also, in agreement with the findings from non-disabled group, the stroke length showed a negative correlation with propulsion velocity for both tests. Stroke time is examined in place of the stroke length, as established from the non-disabled group pilot. The correlation between push frequency and stroke time with propulsion velocity is examined on wheelchair tennis athletes to find the strength of the correlation.

**Table 3:** Push frequency, stroke time, propulsion velocity and RPE during comfortable speed test, with and without racquet.

Comfortable speed test								
Athlete ID	With racquet				Without racquet			
	PF (1/s)	ST (s)	PV (m/s)	RPE	PF (1/s)	ST (s)	PV (m/s)	RPE
1	0.7407	0.38	1.2346	0.0	0.6920	0.62	0.8651	0.0
	0.9195	0.40	1.1494	0.0	0.7790	0.40	0.9737	0.0
	0.9238	0.38	1.1547	0.0	0.7087	0.74	0.7874	0.0
2	0.9036	0.50	1.0040	0.0	0.7890	0.66	0.9862	0.0
	0.9579	0.52	0.9579	0.0	0.8097	0.52	1.0121	0.5
	0.8991	0.48	0.9990	0.0	0.9004	0.52	1.2005	2.0
3	0.4745	0.40	1.1862	0.0	0.9309	0.44	1.3298	0.0
	0.7487	0.48	1.0695	0.0	0.8621	0.52	1.2315	0.0
	0.7463	0.46	1.0661	0.0	0.8759	0.46	1.4599	0.0
4	0.9188	0.42	1.5314	0.0	0.8319	0.46	1.6639	0.0
	0.7924	0.40	1.5848	0.0	0.8562	0.36	1.7123	0.0
	0.9967	0.38	1.6611	1.0	0.8197	0.36	1.6393	1.0
5	1.0029	0.42	1.4327	0.0	0.9642	0.46	1.3774	0.0
	1.1513	0.40	1.6447	0.0	0.9859	0.46	1.4085	0.0
	1.1024	0.42	1.5748	0.0	0.8827	0.50	1.2610	0.5
6	0.8345	0.42	1.3908	3.0	0.5888	0.58	0.9814	2.0
	0.7022	0.40	1.4045	3.0	0.6739	0.56	1.3477	2.0
	0.8451	0.42	1.4085	2.0	0.6579	0.50	1.3158	1.0
7	0.9852	0.44	1.6420	0.0	0.8361	0.42	1.6722	0.0
	0.9934	0.40	1.6556	1.0	0.7962	0.32	1.5924	0.0
	1.0169	0.40	1.6949	1.0	0.8834	0.38	1.7668	1.0
8	1.1538	0.32	1.9231	1.0	0.9390	0.36	1.5649	0.0
	1.1765	0.34	1.9608	1.0	1.0438	0.32	2.0877	0.0
	0.9862	0.30	1.9724	1.0	0.8977	0.30	1.7953	0.0
9	0.6791	0.30	1.6978	1.0	0.6849	0.32	1.7123	3.0
	0.8787	0.30	1.7575	1.0	0.9346	0.28	1.8692	3.0
	0.8929	0.28	1.7857	1.0	1.0288	0.26	2.0576	2.0
Mean ± SD	0.90 ± 0.16	0.40 ± 0.06	1.46 ± 0.31	1 ± 1	0.83 ± 0.11	0.45 ±0.12	1.43 ± 0.35	1 ± 1

*PF Push frequency (push/s), *ST* Stroke time (s), *PV* Propulsion velocity (m/s).

**Table 4:** Push frequency, stroke time, propulsion velocity and RPE during sprint test, with and without racquet.

Sprint test								
Athlete ID	With Racquet				Without Racquet			
	PF (1/s)	ST (s)	PV (m/s)	RPE	PF (1/s)	ST (s)	PV (m/s)	RPE
1	1.5957	0.24	2.6596	0.0	1.5625	0.28	2.6042	2.0
	1.6173	0.24	2.6954	0.5	1.5789	0.24	2.6316	2.0
	1.6043	0.30	2.6738	1.0	1.5831	0.30	2.6385	2.0
2	1.5504	0.22	2.5840	3.0	1.7143	0.24	2.8571	5.0
	1.9284	0.24	2.7548	3.0	1.7341	0.22	2.8902	5.0
	1.6304	0.24	2.7174	3.0	2.0000	0.26	2.8571	4.0
3	1.6760	0.26	2.7933	2.0	1.7804	0.28	2.9674	3.0
	1.9074	0.24	2.7248	0.5	1.8576	0.26	3.0960	4.0
	1.8817	0.24	2.6882	2.0	1.5152	0.28	3.0303	4.0
4	1.7751	0.30	1.9724	3.0	1.8141	0.28	2.2676	3.0
	1.4141	0.32	2.0202	3.0	1.8913	0.26	2.3641	3.0
	1.4056	0.30	2.0080	3.0	1.8059	0.26	2.2573	3.0
5	2.2472	0.26	2.8090	1.0	2.3881	0.26	2.9851	0.0
	2.2727	0.24	2.8409	1.0	2.4242	0.24	3.0303	0.5
	2.2792	0.26	2.8490	2.0	1.7143	0.22	2.8571	1.0
6	1.5119	0.30	2.1598	5.0	1.2579	0.32	2.0964	4.0
	1.2346	0.32	2.0576	5.0	0.9901	0.30	1.9802	4.0
	1.4170	0.30	2.0243	4.0	1.2000	0.32	2.0000	3.0
7	1.1834	0.34	1.9724	4.0	1.3015	0.34	2.1692	3.0
	1.2371	0.34	2.0619	4.0	1.2766	0.34	2.1277	4.0
	1.0753	0.40	1.7921	4.0	1.1364	0.32	2.2727	3.0
8	1.5873	0.28	2.2676	3.0	1.3021	0.28	2.6042	1.0
	1.4634	0.28	2.4390	3.0	1.2920	0.28	2.5840	1.0
	1.4528	0.30	2.4213	3.0	1.2626	0.28	2.5253	1.0
9	1.5801	0.28	2.2573	3.0	1.2500	0.26	2.5000	5.0
	1.5419	0.28	2.2026	3.0	1.1962	0.24	2.3923	5.0
	1.6842	0.28	2.1053	3.0	1.2019	0.26	2.4038	5.0
Mean ± SD	1.62 ± 0.31	0.28 ± 0.04	2.39 ± 0.34	3 ± 1	1.5 ± 0.36	0.27 ± 0.03	2.55 ± 0.34	3 ± 2

*PF Push frequency (push/s), *ST* Stroke time (s), *PV* Propulsion velocity (m/s).

**Table 5:** Push frequency, stroke time, propulsion velocity and RPE during round trip sprint test, with and without racquet.

Round trip sprint test								
Athlete ID	With racquet				Without racquet			
	PF (1/s)	ST (s)	PV (m/s)	RPE	PF (1/s)	ST (s)	PV (m/s)	RPE
1	1.3631	0.24	2.4783	2.0	1.5068	0.26	2.7397	3.00
	1.3547	0.28	2.4631	2.0	1.3055	0.26	2.6110	4.00
	1.3415	0.28	2.4390	3.0	1.5789	0.26	2.6316	4.00
2	1.4286	0.22	2.5974	4.00	1.4865	0.24	2.7027	7.00
	1.5190	0.22	2.5316	3.00	1.5027	0.24	2.7322	7.00
	1.6582	0.24	2.5510	4.00	1.4474	0.24	2.6316	6.00
3	1.5915	0.26	2.6525	3.00	1.4493	0.26	2.8986	4.00
	1.4512	0.26	2.6385	2.00	1.4286	0.28	2.8571	5.00
	1.6000	0.26	2.6667	3.00	1.3986	0.28	2.7972	5.00
4	1.3592	0.30	1.9417	4.00	1.3919	0.28	2.1413	3.00
	1.4205	0.30	1.8939	4.00	1.3728	0.28	2.1119	4.00
	1.4591	0.28	1.9455	5.00	1.4629	0.28	2.0899	5.00
5	1.8301	0.24	2.6144	2.00	1.8445	0.24	2.6350	0.50
	1.9036	0.24	2.5381	3.00	1.8494	0.26	2.6420	1.00
	1.6839	0.26	2.5907	3.00	1.5936	0.22	2.6560	2.00
6	1.3015	0.30	2.1692	7.00	0.9626	0.34	2.1390	5.00
	1.2141	0.30	2.2075	7.00	1.1390	0.30	2.2779	5.00
	1.1628	0.34	2.1142	7.00	1.1375	0.36	2.0683	5.00
7	1.1407	0.34	1.9011	4.00	1.1715	0.34	2.1299	4.00
	0.9839	0.34	1.7889	5.00	1.0352	0.34	2.0704	5.00
	0.9461	0.38	1.8921	4.00	1.0173	0.34	2.0346	5.00
8	1.2422	0.28	2.0704	4.00	2.1254	0.28	2.1254	2.00
	1.1044	0.28	2.0080	4.00	2.3895	0.28	2.3895	2.00
	1.1089	0.26	2.0161	4.00	2.2396	0.28	2.2396	2.00
9	1.2172	0.32	1.8727	5.00	1.4689	0.28	2.2599	7.00
	1.2862	0.28	2.1436	5.00	1.3100	0.20	2.1834	7.00
	1.2500	0.28	2.0833	5.00	1.3575	0.22	2.2624	7.00
Mean ± SD	1.36 ± 0.23	0.28 ± 0.04	2.25 ± 0.30	4 ± 1	1.48 ± 0.35	0.28 ± 0.04	2.41 ± 0.29	4 ± 2

*PF Push frequency (push/s), *ST* Stroke time (s), *PV* Propulsion velocity (m/s).

**Fig. 2:**
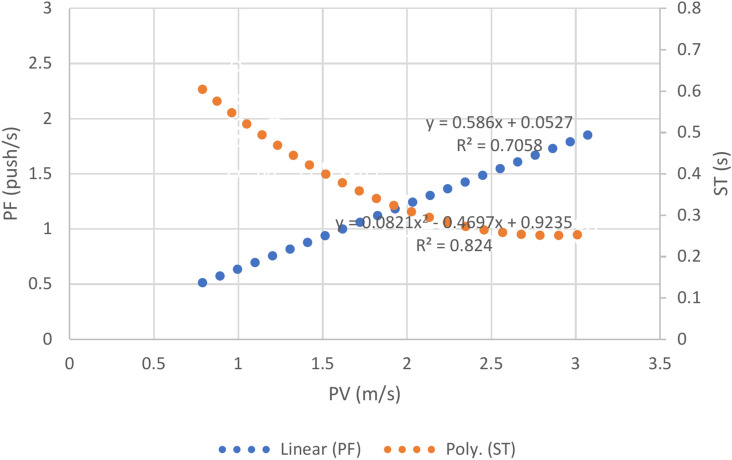
The relationship between push frequency and stroke time on propulsion velocity.

The correlation between push frequency and velocity was significantly positive (*r* = 0.840, *p* < 0.001). However, the stroke time and velocity showed a significantly negative correlation, (r =  − 0.859, p < 0.001) (Fig. 2). These correlation values are much stronger compared to the non-disabled group’s correlation, but with similar trend.

Table 6 shows the correlation of push frequency and stroke length with the athletes’ RPE. Based on the table shown, it can be clearly seen that RPE has a positive correlation with push frequency and propulsion velocity, but negative correlation with stroke time.

**Table 6:** Correlation of push frequency, stroke length and propulsion velocity with Rate of perceived exertion (RPE).

Parameters	Rate of perceived exertion (RPE)	
	Pearson correlation	p-value
Push Frequency (push/s)	0.266	< 0.001
Stroke Time (s)	− 0.477	< 0.001
Velocity (ms^-1^)	0.438	< 0.001

### Performance of elite vs novice and injury type

**Fig. 3:**
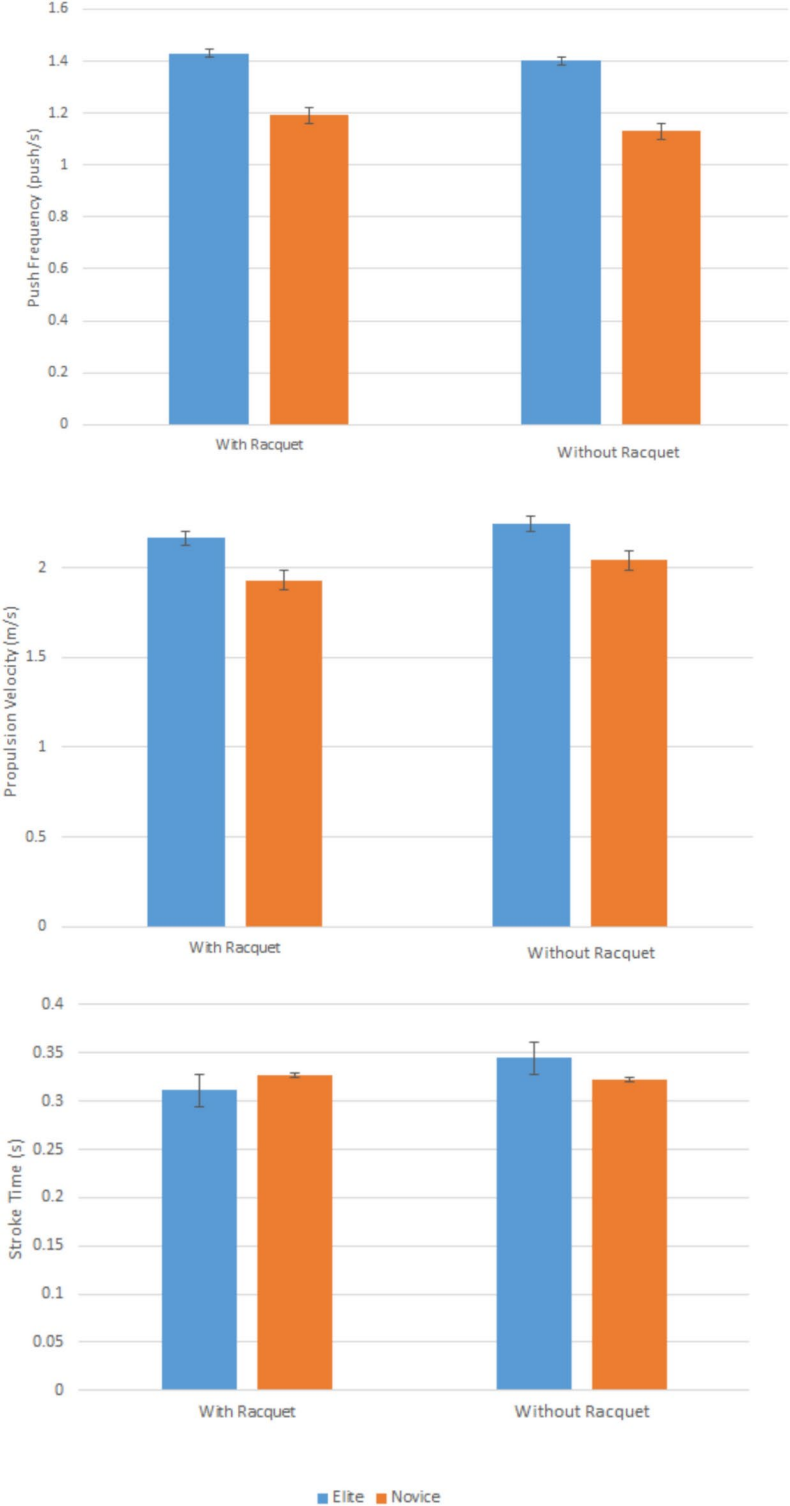
(top) Push frequency (middle) Propulsion velocity, and (bottom) Stroke time; with and without racquet, of elite and novice athletes.

Some additional observations were made from this study. Elite wheelchair tennis athletes have a higher mean push frequency and propulsion velocity compared to novice athletes (Fig. 3). This is consistent with a previous study that also reported that players with higher ranking have higher push frequency and shorter stroke time, thus cover more distance compared to lower ranking players during a match^16^. This was also observed in higher ranking basketball players who performed better propulsion compared to low-ranking players, thus scoring higher in a match^17^. At the same time, the mean push frequency for all athletes holding a racquet is comparatively higher than athletes without a racquet. It was observed that the maximal speed of wheelchair tennis athletes is reduced when holding the racquet, with the speed in the pushes is reduced significantly^18^. On the other hand, the mean stroke length for athletes without racquet seems to be higher as compared to the athletes with racquet in the novice group but not the elite group. However, due to the mixed stroke time effect when racquets were involved, the condition leads to higher wheelchair velocity among elite and novice athletes when not holding racquets during the propulsion even with less push frequency applied. Rietveld et al*.* had the left and right wheels instrumented separately for independent velocity measurements corresponding to the side of the arms holding the racquet. As results, they were able to identify that holding tennis racquets was important in contributing to wheelchair maneuverability in tennis wheelchair players, in rotational velocities and linear velocities^19^.

The study also compared the performance and propulsion behavior of the athletes based on their injury type (Table 1). Based on Table 7, amputees athletes tend to have a higher mean push frequency and propulsion velocity, with lower stroke time, compared to athletes with spinal cord injury. Athletes with spinal cord injury have a higher mean stroke time than the amputee athletes. This may be explained by the compromised trunk control among athletes with spinal cord injury in a previous study^20^. The difference however was not statistically tested as the sample size was too small to validly compare, and their overall performance may vary according to the athletes’ experience and gained skills.

**Table 7:** Push frequency, stroke time and propulsion velocity according to injury type.

	Push frequency (push/s)	Stroke time (s)	Propulsion Velocity (m/s)
Spinal Cord Injury	1.25 ± 0.40	0.48 ± 0.10	2.02 ± 0.49
Amputee / Limb Differences	1.30 ± 0.36	0.46 ± 0.09	2.19 ± 0.66

## Discussion

This study investigated the correlation of professional athletes’ mechanical input during propulsion with their wheelchair propulsion velocity on their actual training environment, i.e., a tennis hardcourt.

Based on the Pearson correlation results, it can be concluded that there is a significant positive relationship between push frequency and velocity for both comfortable speed test and sprint test, while the correlation between stroke time and velocity represents a negative relationship. The analytical outcome shows that the push frequency has positive correlation with the propulsion velocity on wheelchair. Van Drongelen et al. reported a similar outcome in their research on the effect of workload setting on the wheelchair propulsion technique using SMARTWheel and treadmill^10^. They reported that push speed decreases with push frequency, however insisted that the study might be limited in the aspect on type of participants as all the participants are from able-bodied categories with no experience using wheelchairs. The inexperience of wheelchair users and no restriction due to disability might cause similar effect on the accuracy of the result obtained^21^.

The higher the push rate, the higher the acceleration per push which subsequently results in higher propulsion velocity. Goosey-Tolfrey and Moss indicated that the maximum velocity decreases when holding a racquet during propulsion compared to without holding a racquet, significantly during the first three pushes^22^. The maximum number of pushes reflected by push frequency is directly proportional to sprint time^1^, and the propulsion velocity might be affected by the interface technique between the pushrim and the hand. Less efficient interface between hand and pushrim may affect the friction in the direction of propulsion and also increase the power loss during coupling between the pushrim and hand^23^.

Stroke time, measured replacing stroke length as the time length of one complete push, can influence the propulsion in greater scale than stroke length of two consecutive pushes. Van Drongelen and colleagues reported that push time have a strong negative correlation with propulsion velocity^10^. Sauret and team examined this effect on non-disabled athletes with self-selected comfortable speed with few trial data^4^. There is a strong negative correlation between push frequency and stroke time. Our study verified all these findings in professional wheelchair athletes performing in their actual training environment.

Low push frequency results in lower blood lactate concentration^9^. A previous study reported that inspiratory muscle fatigue can contribute to limited blood flow and perfusion in the upper limb muscles that are responsible for wheelchair propulsion. In addition, it does suggest that Inspiratory muscle training could improve muscle endurance, thus lowering the RPE rate in wheelchair tennis players^24^. RPE rate during the propulsion with holding the racquet is higher compared to without holding the racquet. This contradicts the fact that when holding the racquet, higher peak forces applied on the shoulder which can influence their exertion level. The athletes find it harder to propel the wheelchair while holding the racquet and experience more power loss after the push is made. In addition, the RPE rating can be affected by the mood of the user as exercise mood can influence the RPE output from the participants. The study shows that participants who with a positive mood can show higher RPE, while participants with a negative mood will show a lower RPE^25^.

### Study limitations

The investigation on wheelchair athletes were carried out only on hard court surfaces. The players might have to play in clay court surfaces when they are taking part in international competitions. The propulsion pattern of the wheelchair might vary from that of hard court as the rolling resistance might be higher in the clay court.

The relatively small number of participants for this experimental study is also one limitation of our findings as the exposure level for wheelchair tennis is still low in the country. Additionally, the research is based on short term duration rather than a long-term study where further outcomes could be measured in order to understand the fatigue effect or training effect to the players propulsion maneuverability. In addition, as the athletes used their own wheelchairs, the researchers do not have access to their model numbers.

In terms of effort measurement, the exertion level was measured in RPE which is a subjective measure. A more quantitative exertion level measurement parameter such as heart rate or VO_2_ can provide more accurate cardiorespiratory responses of the participants. Future studies could consider heart rate measurement and oxygen uptake level to be included as a measure of the physiological and cardiorespiratory responses rather than depending on RPE test alone. Motion sensors can be integrated with the tennis wheelchair to obtain more accurate propulsion velocity and other measures of maneuverability parameters. The inclusion of the parameter of body posture during wheelchair propulsion can also be examined as the user controllable factor effecting wheelchair mobility.

More can be investigated about their hardcourt maneuverability, including rotational speed and acceleration^26^ or their hand trajectory during propulsion technique^15^. All these maneuvers were not investigated as it is beyond the scope of this study in this study, and could be picked up by future research. This may include instrumenting the wheels with accelerometers to recognize ‘push’ and ‘stop’ maneuver^27^ in order to monitor the wheelchair athletes’ performance during competitions and trainings.

## Conclusion

Various parameters contributing towards wheelchair mobility such as push frequency, stroke length and propulsion velocity were tested on different types of propulsion surface. The push frequency is found to have positive correlation with propulsion velocity (r = 0.478 in non-disabled group and 0.840 in wheelchair tennis athletes’ group, p < 0.05) which indirectly represents the wheelchair mobility. The stroke length is negatively correlated with propulsion velocity in both groups, also with significantly stronger correlation in the wheelchair tennis athletes’ group (r = -0.4 in non-disabled group and r = -0.859 in wheelchair tennis athletes’ group, p < 0.05). More studies can be done by using inertial sensors, to analyze more specific movements in wheelchair tennis players. Researchers have stated that inertial sensors can be integrated in the methodology to analyze complex movements of wheelchair athletes, providing more precise results on wheelchair maneuvers^26^.

In conclusion, the wheelchair propulsion velocity during the wheelchair tennis competition can be enhanced with higher push frequency along with lower stroke time. The athletes could train propelling with higher number of pushes and the period of each push should be shorter to obtain higher propulsion velocity.

## Data Availability

The main data generated or analysed during this study are included in this published article. The datasets used and/or analysed during the current study is available from the corresponding author on reasonable request.
